# Modulation of chemokine and chemokine receptor expression following infection of porcine macrophages with African swine fever virus

**DOI:** 10.1016/j.vetmic.2012.11.027

**Published:** 2013-03-23

**Authors:** Emma Fishbourne, Charles C. Abrams, Haru-H. Takamatsu, Linda K. Dixon

**Affiliations:** The Pirbright Institute, Ash Road, Pirbright, Woking, Surrey GU24 0NF, UK

**Keywords:** African swine fever, Chemokines, Immune evasion, Transcription

## Abstract

African swine fever virus (ASFV) is the only member of the *Asfarviridae*, a large DNA virus family which replicates predominantly in the cytoplasm. Most isolates cause a fatal haemorrhagic disease in domestic pigs, although some low virulence isolates cause little or no mortality. The modulation of chemokine responses following infection of porcine macrophages with low and high virulence isolates was studied to indicate how this may be involved in the induction of pathogenesis and of effective immune responses. Infection with both low and high virulence isolates resulted in down-regulation of mRNA levels for chemokines CCL2, CCL3L, CXCL2 and chemokine receptors CCR1, CCR5, CXCR3, CXCR4 and up-regulation in expression of mRNAs for CCL4, CXCL10 and chemokine receptor CCR7. Levels of CCL4, CXCL8, CXCL10 mRNAs were higher in macrophages infected with low virulence isolate OURT88/3 compared to high virulence isolate Benin 97/1. Levels of CXCL8 and CCL2 protein were significantly reduced in supernatants from macrophages infected with Benin 97/1 isolate compared to OURT88/3 and mock-infected macrophages. There was also a decreased chemotactic response of donor cells exposed to supernatants from Benin 97/1 infected macrophages compared to those from OURT88/3 and mock-infected macrophages. The data show that infection of macrophages with the low virulence strain OURT88/3 induces higher expression of key inflammatory chemokines compared to infection with high virulence strain Benin 97/1. This may be important for the induction of effective protective immunity that has been observed in pigs immunised with the OURT88/3 isolate.

## 1 Introduction

African swine fever (ASF) is a fatal haemorrhagic disease of domestic pigs caused by a large, cytoplasmic DNA virus, African swine fever virus (ASFV). ASFV is currently the only member of the *Asfarviridae* family (Dixon et al., 2005). The natural reservoir hosts of ASFV are the warthog (*Phacochoerus africanus*) and bush pig (*Potamchoerus porcus*), which show few if any clinical signs of disease, although they can be persistently infected (Oura et al., 1998a). ASFV isolates differ in virulence and include, in addition to highly virulent isolates, isolates of moderate virulence from which up to 50% of pigs survive infection and low virulence isolates which cause few clinical signs. Low virulence isolates, such as OURT88/3 or NHVP68 (Boinas et al., 2004; Leitao et al., 2001), can induce protection against challenge with related virulent viruses.

ASF is characterised by apoptosis of bystander lymphocytes. Virulent isolates cause severe tissue destruction and cell depletion in lymphoid tissue (Carrasco et al., 1996; Oura et al., 1998b). ASFV primarily infects macrophages and soluble mediators produced by these cells have been implicated in ASFV pathogenesis. These mediators include tumour necrosis factor (TNF)α which has been shown to be elevated in the serum and tissues of pigs infected with virulent ASFV isolates (del Moral et al., 1999; Salguero et al., 2008).

ASFV encodes between 150 and 167 proteins, a number of which have been shown to modulate host immune responses (Dixon et al., 2004; Tulman et al., 2009). Proteins have been identified that inhibit interferon induction, host transcription factor activation, stress responses and apoptosis. The ASFV gene A238L codes for a protein that inhibits transcriptional activation of a range of host immunomodulatory genes, including pro-inflammatory cytokines and chemokines by inhibiting activation of NFκB, calcineurin phosphatase(Miskin et al., 1998; Powell et al., 1996) and the host transcriptional co-activator p300. This latter effect seems specific to transcription factors that act through the N-terminal but not C-terminal domain of p300 (Granja et al., 2009). An ASFV Toll like receptor (TLR)3 inhibitor, encoded by the ASFV gene I329L has been identified (de Oliveira et al., 2011) which could reduce transcription of genes through its effect on the pattern recognition receptors (PPRs) and resultant downstream signalling pathways. In addition deletion of 6 members of multigene family 360 (MGF360) and 2 of multigene family 530 (MGF530), from a virulent isolate Pr4, was shown to result in an increased type I IFN response and induction of IFN stimulated genes (ISGs). This suggests that these genes inhibit type I IFN induction and possibly IFN activated pathways (Afonso et al., 2004). Macrophages, the main target cells for ASFV replication, produce a range of inflammatory mediators including chemokines. Chemokines contribute to induction of pathology and protective immunity (Sallusto et al., 2000; Wack et al., 2011). Inflammatory chemokines promote the migration of leucocytes to an injured or infected cell, activate cells to raise an immune response and start wound healing (Baggiolini, 1998; Johnston and Butcher, 2002; Laing and Secombes, 2004; Moser and Loetscher, 2001). The importance of chemokines in the antiviral response is emphasised since other large DNA viruses have developed strategies to evade chemokine responses. These include virus-encoded proteins that mimic chemokines and their receptors or secreted proteins that are able to bind host chemokines. These proteins function to modulate the normal function of the host chemokine system (Alcami and Lira, 2010). Viral versions of chemokines may function as antagonists, agonists or facilitate dissemination and growth of the virus.

In the present study the effect of ASFV isolates, varying in virulence on the host chemokine system was compared in macrophages following infection *in vitro*, since these are the main cell type infected *in vivo*. The aim was to better understand the differences in response in the infected macrophages rather than in bystander cells and thus to identify how ASFV infection may initially modulate the host chemokine response. To achieve this, primary pig macrophages isolated from blood were infected at high multiplicity to obtain infection rates of greater than 90%. The mRNA levels were measured for a number of inflammatory chemokines and chemokine receptors known to be expressed in macrophages. The induction of chemotactic substances in supernatants from infected macrophages was also studied.

## 2 Materials and methods

### 2.1 Preparation and culture of porcine macrophages

Blood-derived macrophages were obtained from whole blood by incubating the blood with 6% Dextran for 30 min at 37 °C to sediment red blood cells with subsequent lysis of any contaminating red blood cells remaining using ammonium chloride. Remaining leucocytes were then washed and re-suspended in DMEM plus Hepes containing 30% porcine serum and plated in 12-well culture dishes. Cells were cultured at 37 °C and 5% CO_2_ for 2 h then non-adherent cells were removed and cells were further cultured for 48 h before washing 3 times to remove non-adherent cells.

### 2.2 Virus isolates, titrations and infections

ASFV isolates used for infections were low virulence OURT88/3 isolate (Boinas et al., 2004) and the high virulence isolate Benin 97/1 (Chapman et al., 2008) which both belong to genotype I. These isolates were chosen because complete genome sequences are available and the proteins encoded are very closely related. Differences between the genomes include deletions of 5 members of MGF360 and 2 members of MGF530 from close to the left genome end and disruptions of genes EP402R and EP153R in the genome of the OURT88/3 isolate. These encode a CD2-like protein and a C-type lectin (Chapman et al., 2008).

These isolates were propagated on porcine bone marrow cells and purified (Zhang et al., 2006). Stocks were titrated by haemadsorption (Malmquist, 1960). Blood-derived macrophages were infected at a multiplicity of infection of 5 by incubating the cells for 2 h in serum free media. Mock-infected cells were treated in the same way using virus-free media. At 16 h post-infection, cell supernatants or lysates were recovered. Cell supernatants were centrifuged to remove any cellular debris and used in chemotaxis or ELISA assays. RNA was extracted from lysates as described below. Infection rates were measured by fixing cells in 4% paraformaldehyde, permeabilising with 0.2% Triton-X-100 and staining with anti-ASFV p30 and VP72 monoclonal antibodies C18 and 4H3 at a dilution of 1 in 10 (Cobbold and Wileman, 1998; Stefanovic et al., 2005), followed by Alexa-Fluor conjugated anti-mouse IgG1 antibodies (Invitrogen) at a concentration of 1 in 200 and visualised using a Leica TCSSP2 confocal microscope.

### 2.3 RNA preparation and quantitative reverse transcription PCR

RNA was isolated, from infected or mock-infected cells harvested at 16 h post-infection, using Qiagen RNAeasy Blood mini kits with Qiagen Shredders and eluted in a 50 μl volume. Contaminating DNA was removed using Turbo DNAse (Ambion) and RNA quality was assessed using a Bioanalyser 2100 (Agilent) and the quantity was measured using a nanodrop. Two hundred and fifty ng of total RNA was reverse transcribed into cDNA using Superscript III (Invitrogen) and anchored oligo dT primer. Quantitative PCR was carried out using SYBR Green I (Sigma) and 10 μl volume reactions and read using the Mx3005P Stratagene thermal cycler. Primers and reaction conditions are given in Supplementary Table 1. Reactions were carried out in duplicate in experiments using cells from 9 different outbred pigs. Normalisation was carried out relative to the amount of RNA added to the quantitative reverse transcription PCR (qRT-PCR) reactions and fold changes were calculated relative to the samples from mock-infected cells harvested at 16 h post-infection. A general linear model was used to analyse the results using Minitab 16 and results were grouped using the Tukey method.

### 2.4 ELISA assays

The amount of IFNγ, CCL2 and CXCL8 in supernatants from infected and mock-infected cells was determined using commercially available kits (Kingfisher) according to the manufacturer's instructions.

### 2.5 Chemotaxis assays

Virus was removed from supernatants from cell cultures using 300,000 molecular weight cut-off PES Vivaspin filter centrifugation tubes (Sartorius). PBLs from uninfected donor pigs were obtained using the method described above, without the selection for adherent cells and subsequent culturing. Migration of these cells to supernatants from infected and mock-infected cultures was measured using 30 μl 96-well Chemotaxis Chambers (Neuroprobe), with a 5 μm pore membrane. CCK-8 (Donji) was used to stain the PBLs which migrated across the membrane. PMN cells were isolated as described for the PBLs, with an additional purification on Histopaque gradients, and used in flow cytometry assays to measure changes in forward scatter which result from cell activation by chemokines.

## 3 Results

### 3.1 Measurement of changes in mRNA levels for chemokine and chemokine receptor genes following infection

Blood derived macrophages were infected at a high multiplicity with the high virulence Benin 97/1 or low virulence OURT88/3 isolates. Infection rates were assessed at a late stage, 16 h post-infection by staining with antibodies against ASFV proteins p72 and p30 and observation by confocal microscopy. Typical infection rates of between 90 and 100% were observed in infections of cells from 9 different pigs with each isolate. Mean viral titres recovered from infections of cells from 6 pigs were 6.46 Log_10_ TCID_50_ml^−1^ from cells infected with the OURT88/3 isolate and 6.25 Log_10_ HAD_50_ml^−1^ from the cells infected with the Benin 97/1 isolate. Thus the infection and replication rates of the OURT88/3 and Benin 97/1 isolates were very similar.

**Fig. 1:**
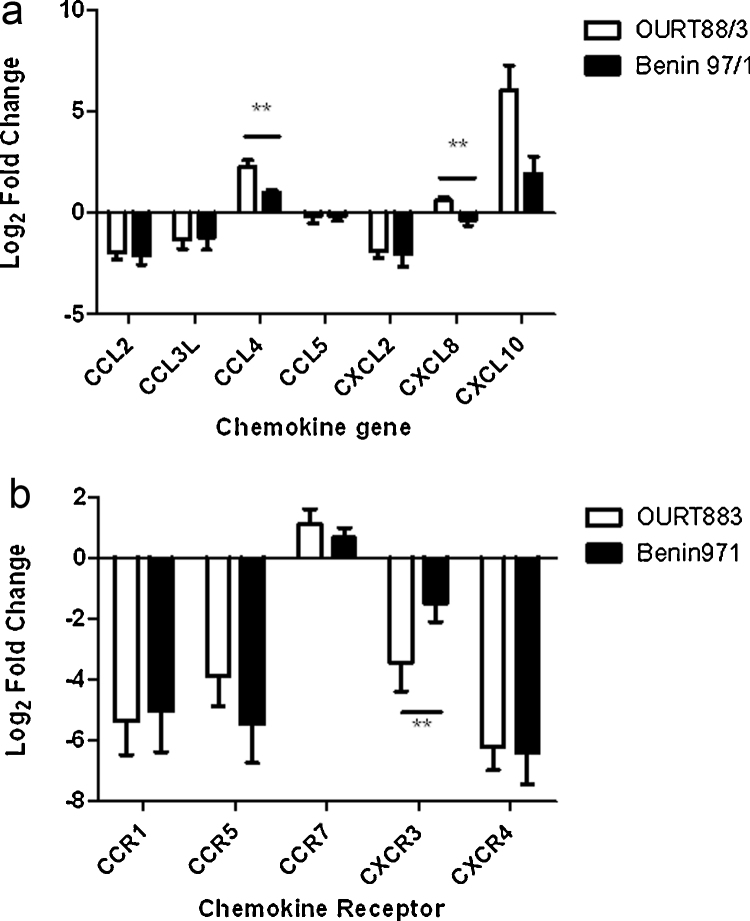
Comparison of mRNA levels for chemokine and chemokine receptor genes following infection of porcine macrophages *in vitro* with high virulence Benin 97/1 or low virulence OURT88/3 isolates. Porcine blood derived macrophages were infected at a multiplicity of 5 with ASFV isolates Benin 97/1 and OURT88/3 isolates or were mock-infected. At 16 h post-infection RNA samples were harvested and levels of mRNAs for chemokine (panel A) or chemokine receptor (panel B) genes were estimated by quantitative reverse transcriptase PCR. Log_2_ fold changes of mRNAs relative to mock infected samples are shown (*y* axis). Results from infections with OURT88/3 isolate are shown in open boxes and from infections with Benin 97/1 as closed boxes. Changes calculated to be significant are indicted by a double asterisk (*p* = −0.009). The mRNAs measured are indicated on the *x* axis and were in panel A, CCL2, CCL3L, CCL4, CCL5, CXCL2, CXCL8, CXCL10 and in panel B, CCR!, CCR5, CCR7, CXCR3 and CXCR4.

Cell supernatants and RNA were harvested at 16 h post-infection. Samples from mock-infected cells, also harvested at 16 h, were used as calibrator samples for gene expression studies. The change in levels of mRNA for porcine chemokine ligands and receptors was estimated by qRT-PCR (Fig. 1a and b). Variation was observed following infections using macrophages derived from different outbred pigs as expected and mean levels of mRNA were calculated. This showed that the mean levels of mRNA for chemokine ligands CCL2, CCL3L1 and CXCL2 were down-regulated by between 2 and 4-fold (CCL2 *p* = 0.011, CCL3 *p* = 0.024, CXCL2 *p* = 0.009) and mRNAs for chemokine receptors CCR1, CCR5, CXCR3 and CXCR4 were down-regulated by between 4 and 12-fold in ASFV infected-macrophages compared to mock-infected controls (CCR1 *p* = 0.006, CCR5 *p* = 0.008, CXCR3 *p* = 0.005, CXCR4 *p* = 0.010). Levels of mRNAs for CCL5 remained stable following infections with both isolates. Levels of CCR7 mRNA were up-regulated by about 2-fold compared to samples from mock-infected cells and levels of mRNAs for CCL4 and CXCL10 were up-regulated by 2 to 4-fold in infected cells in comparison to mock-infected controls.

Data on mRNA levels following infections with different isolates were statistically grouped using the Tukey method and 95% confidence. This showed there was a significant difference in levels of mRNAs in cells infected with the different isolates for CCL4, CXCL8 and CXCR3 and a trend for a difference in levels of mRNA for CXCL10. Levels of mRNA for CCL4 and CXCL8 were significantly up-regulated in the macrophages infected with OURT88/3 isolate compared to those infected with Benin 97/1. The same trend was observed for CXCL10 mRNA and the CXCR3 mRNA level was significantly less down-regulated in the Benin 97/1 infected macrophages compared to OURT88/3 macrophages. No Ct values were recorded for the controls containing no template or no reverse transcriptase.

### 3.2 Levels of CCL2 and CXCL8 protein in supernatants from infected cells

**Fig. 2:**
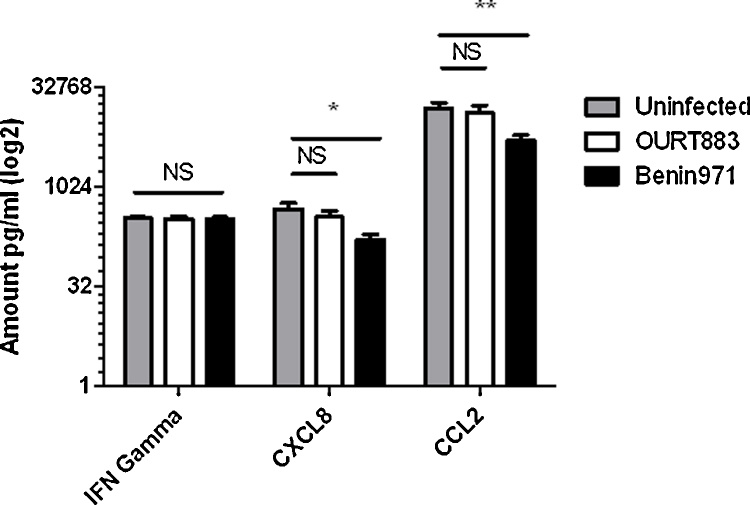
Amounts of IFNγ, CXCL8 and CCL2 in cell supernatants from macrophages mock-infected or infected with ASFV Benin 97/1 or OURT88/3 isolated at 16 h post infection. Porcine blood-derived macrophages were infected, at a multiplicity of 5, with ASFV isolates Benin 97/1, OURT88/3 or mock-infected. At 16 h post-infection cell supernatants were harvested and the amount of IFNγ, CXCL8 and CCL2 present was measured by ELISA. The *y* axis indicates amounts in pg/ml on a log_2_ scale. The *x* axis indicates the different isolates and assays for IFN gamma, CXCL8 or CCL2. Results from infections with OURT88/3 are shown as open boxes, Benin 97/1 as black boxes and from mock-infected cells as grey boxes. Data from infections of cells from 6 different pigs are shown. *Significant difference between groups (*p* = 0.011). **Significant difference between groups (*p* = 0.009). NS indicates not significant differences.

Protein levels of CCL2 and CXCL8 were measured in supernatants from infected macrophages harvested at 16 h post-infection by ELISAs. High levels of CCL2 were detected and lower levels of CXCL8. IFNγ was also measured as a control and very low levels were detected which was as expected since macrophages do not produce IFNγ. The results showed that levels of CCL2 (*p* = 0.000) and CXCL8 (*p* = 0.003) were significantly lower in the cell supernatants from macrophages infected with the virulent Benin 97/1 isolate compared to the low virulence OURT88/3 isolate and mock-infected cells (Fig. 2).

### 3.3 Levels of chemotactic substances in supernatants from infected cells

**Fig. 3:**
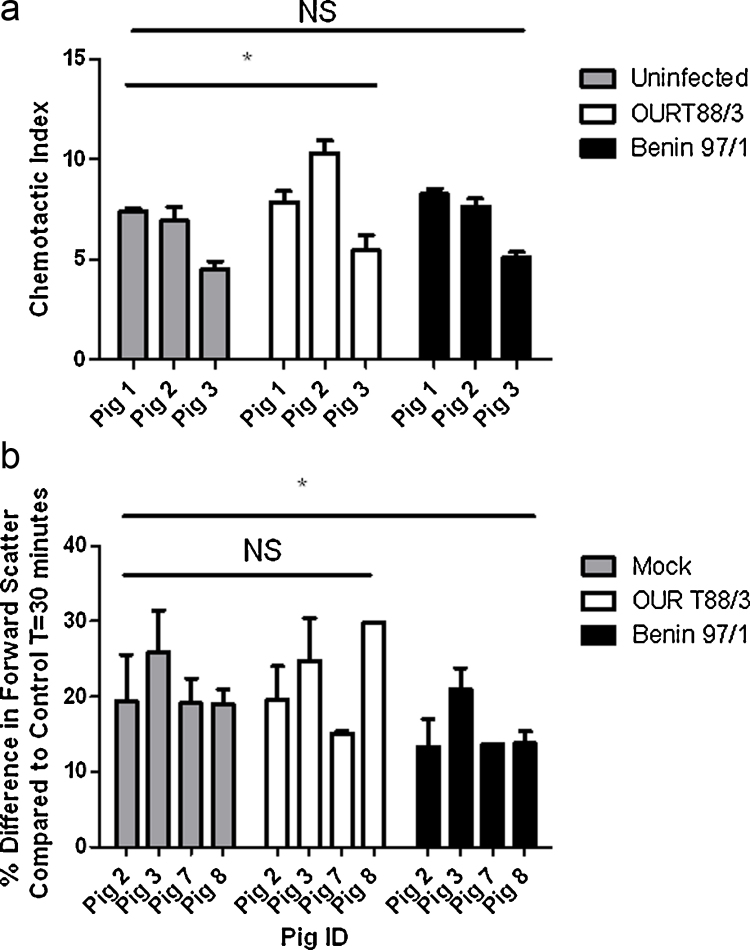
Chemotactic response of donor cells from uninfected pigs to cell supernatants from macrophages either mock infected or infected with ASFV isolates Benin 97/1 or OURT88/3. Blood derived macrophages were infected with ASFV isolates Benin 97/1 or OURT88/3 or mock-infected. At 16 h post-infection cell supernatants were harvested and the chemotactic substances present were measured by (A) migration of PBLs from uninfected donor pigs using a 96-well neuroprobe assay or (B) by measuring by flow cytometry the shape change induced in PMN cells from uninfected donor pigs. In panel A the chemotactic index is shown on the *x* axis and the response of donor cells from 3 uninfected pigs (labelled 1, 2, 3) to supernatants from infected cells from 5 different pigs is indicated on the *x* axis. Panel B shows the change in forward scatter of PMN cells on the *y* axis as the percentage difference compared to control cells measured at 30 min after exposure to supernatants from infected or mock-infected cells. The *x* axis indicates measurements of PMNs from different uninfected pigs (2, 3, 7, 8). Responses to supernatants from cells from 5 different pigs infected with OURT88/3 are shown in white boxes, to Benin 97/1 in black boxes and mock-infected cells in grey boxes. The mean and standard errors are shown. **Significant difference between groups (*p* = 0.002). *Significant difference between groups (*p* = 0.012).

Assays were carried out to determine total levels of chemotactic substances in supernatants from infected and mock-infected cells. Virus was removed from supernatants by filter centrifugation. Migration of PBLs from 3 uninfected donor pigs to a 40% dilution of supernatants, filtered to remove virus, from macrophages from 5 different pigs, either mock-infected or infected with the different isolates of ASFV, was measured. All chemotaxis assays were carried out in quadruplicate (Fig. 3a and b). The trend observed was that more cells migrated towards samples from the macrophages infected with the non-virulent OURT88/3 isolate compared to samples from the mock-infected and Benin 97/1 isolate infected cells, indicating a higher concentration of chemotactic substances was present in the supernatants from OURT88/3 infected macrophages.

In another assay flow cytometry was used to measure changes in forward scatter which would result from cell activation by chemokines. Cell migration and a change in forward scatter (FSC) were assessed following exposure to cell supernatants, purified by filtration to remove virus, from infected and mock-infected cells. Analysis using a general linear model and the Tukey method with 95% confidence showed there was a significant reduction in forward scatter in PMNs exposed to supernatants from Benin 97/1 infected macrophages compared to the PMNs exposed to supernatants from mock-infected and OURT88/3 infected macrophages (Fig. 3B). This supports the conclusion that less chemotactic substances were present in supernatants from the Benin 97/1 infected cells compared to those from OURT88/3 infected or mock-infected cells.

## 4 Discussion

We investigated changes in expression of proinflammatory chemokines and chemokine receptors in macrophages infected *in vitro* with a high and a low virulence ASFV isolate to gain insights into how the virus manipulates the host chemokine response and predict the potential relevance *in vivo*. The results showed that mRNAs for a number of chemokines and chemokine receptors were either down-regulated or not significantly altered in macrophages infected *in vitro* with ASFV, compared to mock-infected cells, at 16 h post-infection or treatment. Those chemokines which had reduced levels of mRNAs in infected cells compared to mock-infected included CCL2, CCL3L, CCL5, CXCL2. However, levels of mRNAs for some chemokines, including CCL4 and CXCL10, were up-regulated compared to mock-infected cells at this time point following infection. This up-regulation was significantly lower in cells infected with high virulence Benin 97/1 compared to low virulence OURT88/3 isolate. CXCL8 mRNA levels were up-regulated in cells infected with OURT88/3 isolate and significantly higher than in cells infected with Benin97/1 isolate. Levels of mRNA for chemokine receptor CCR7 were also slightly increased in infected cells compared to mock-infected cells. In contrast levels of mRNAs for chemokine receptors CCR1, CCR5, CXCR3 and CXCR4 were significantly reduced. The CXCR3 mRNA levels were also significantly lower in cells infected with OURT88/3 compared to Benin 97/1 isolates.

The mechanisms leading to these changes in transcription levels are not known. Since ASFV encodes at least one protein, A238L, which prevents transcriptional activation of a broad range of host immunomodulatory genes, expression of the A238L protein is likely to be involved. Analysis of the sequences of the OURT88/3 and Benin 97/1 isolate genomes identified 5 members of MGF 360 and 2 of MGF 530 which were deleted from a position near the left genome end of the OURT88/3 isolate (Chapman et al., 2008). Deletion of orthologs of these MGF 360 and MGF 530 genes from the genome of Pr4 virulent virus increased levels of type I IFNs secreted from infected macrophages and the induction of type I IFN response genes (Afonso et al., 2004). As predicted from the study reported by Afonso et al. (2004), we observed higher transcript levels for IFN β in OURT88/3 compared to Benin 97/1 infected macrophages (Zhang, Goatley, Dixon unpublished results). Since CXCL10 is induced by type I and type II IFNs the higher mRNA levels for this chemokine in cells infected with OURT88/3 isolate may be as a consequence of increased type I IFN induction in these infections.

In a previous study (Zhang et al., 2006), we used a porcine microarray to analyse changes in mRNA levels following infection of alveolar macrophages with a virulent isolate Malawi LIL20/1. An increase in mRNA levels for a number of host immunomodulatory genes, including chemokines, CCL3, CCL5 and CXCL2 was observed at 4 h post-infection compared to mock-infected cells and a reduction to similar levels as in mock-infected cells at 16 h post-infection (Zhang et al., 2006). We proposed that the early increase in mRNA levels resulted from activation of signalling pathways during virus binding and entry and that expression of virus proteins may modulate the host gene expression at later times. In the current study we analysed changes in mRNA levels at the late time point 16 h post-infection, to increase the chances of observing differential effects on host mRNAs resulting from the expression of the gene complement of each virus isolate. In the current study we observed lower levels of mRNAs for CCL2, CCL3 and CXCL2 relative to mock-infected cells at 16 h compared with similar levels to mock in the previous study (Zhang et al., 2006). This may result from the different techniques used to measure transcripts, since qRT-PCR provides more accurate measurements than microarray analysis.

Extrapolation of results obtained from infections of macrophages *in vitro* with downstream effects that may occur *in vivo* is difficult. *In vivo* bystander effects on non-infected cell populations would be influenced by the sites and levels of virus replication and other factors released from cells. However, we can make predictions from our *in vitro* studies on the likely consequences on virus pathogenesis and development of immune responses *in vivo*. CCL4 is a pro-inflammatory chemokine attracting mononuclear cells and can directly promote development of IFN γ producing lymphocytes (Borish and Steinke, 2003). Both CCL4 and CXCL10 act as chemoattractants for CD4+ T cells. CCL4 can directly promote development of IFN γ producing TH1 lymphocytes through CCR5 or indirectly by promoting IL-12 secretion from antigen presenting cells. CXCL8 is a major mediator of inflammatory responses and can attract neutrophils, basophils, NK and T cells. The OURT88/3 isolate can induce protection against challenge with related virulent ASFV isolates and this protection is dependent on CD8+ cells and correlated with the induction of high levels of antigen specific IFNγ producing cells (King et al., 2011; Oura et al., 2005). Thus increased mRNA levels of CCL4, CXCL8 and CXCL10 in infected macrophages may contribute to effective induction of protective immunity by this isolate. The limited reagents available to measure protein levels for porcine chemokines restricted our analysis to CXCL8 and CCL2. The levels of protein measured by ELISA were of total quantities that accumulated in cell supernatants by 16 h post-infection. These levels were obviously influenced by changes in mRNA levels and protein expression, stability and secretion during the infection period, rather than just at 16 h post-infection. High levels of CCL2 proteins were present in cell supernatants and were decreased in cells infected with Benin 97/1 compared to OURT88/3 or mock-infected cells. Lower levels of CXCL8 were detected in cell supernatants and significantly less was present in those from Benin 97/1 infected compared to OURT88/3 and mock-infected cells. CCL2 is primarily produced by monocytes and macrophages (Leonard and Yoshimura, 1990) and recruits monocytes to sites of active infection, exerting its effect through its receptor CCR2. CCL2 is up-regulated as monocytes mature into a macrophage phenotype. The higher levels of CCL2 detected in supernatants from macrophages infected with OURT88/3 isolate may result in increased attraction of macrophages to sites of replication. As discussed above increased levels of CXCL8 in supernatants from OURT88/3 infected macrophages may attract neutrophils, basophils, NK cells and T cells. These changes may *in vivo* facilitate virus replication by recruitment of more susceptible macrophages, or enhance virus clearance by recruitment of inflammatory cells. Also recruitment of T cells may enhance induction of adaptive immune response. The reduced levels of CXCL8 in supernatants from macrophages infected with Benin 97/1 compared to OURT88/3 isolates correlates with observed differences in mRNA. Levels of CCL2 mRNA at 16 h post-infection did not differ between cells infected with Benin97/1 and OURT88/3 isolates and were lower than in mock-infected cells. As discussed above levels of CCL2 protein secreted may be influenced by factors other than the mRNA level at 16 h post-infection.

Down-regulation of mRNA levels for certain chemokines and chemokine receptors was observed in infected compared to mock infected-cells. If reflected at the level of expressed proteins, this is likely to reduce the chemotactic signals from infected cells directed to other cells and the ability of infected cells to respond to chemotactic signals. The mRNA levels for chemokine receptors CCR1, CCR5, and CXCR4 were all reduced by 8 to 15-fold in infected compared to mock-infected cells. These act as receptors for chemokines including CCL3, CCL4, CCL5, CCL7, CCL8, CCL14, CCL15, CCXCL12 and this may indicate a reduced capacity of infected macrophages to respond to a broad range of chemotactic signals *in vivo*. Levels of mRNA for CXCR3 were reduced by 4-fold in Benin 97/1 infected cells and by a significantly greater level (about 8-fold) in cells infected with OURT88/3 isolate compared to mock-infected cells. CXCR3 is the receptor for several IFNγ induced chemokines, including CXCL9, CXCL10 and CXCL11, and is usually highly expressed on monocytes and macrophages as well as T cells and NK cells. Down-regulation of the CXCR3 receptor would reduce the activation of pathways dependent on this receptor and the migration of infected cells in response to these chemokines. Possibly this may reduce dissemination of virus *in vivo*. As CCR7 is a lymph node homing receptor, enhanced CCR7 may increase the accumulation of ASFV infected macrophages into lymph nodes or spleen. This may lead to further infections of susceptible macrophages or contribute to the pathology observed in lymph nodes and spleen.

Functional chemotaxis assays showed greater movement of cells from uninfected donor pigs towards supernatants from macrophages infected with the non-virulent OURT88/3 compared to virulent Benin 97/1 isolate indicating greater levels of chemotactic substances were secreted from the OURT88/3 isolate infected macrophages. Forward scatter of PMNs, indicating cell activation, was also reduced when these cells were exposed to supernatants from Benin 97/1 infected compared to either mock-infected or OURT88/3 isolate infected cell supernatants. PMNs are primarily activated by CXCL8. mRNA levels for CXCL8 in cells and CXCL8 protein in cell supernatants were both reduced following Benin 97/1 infection compared to either mock-infection or infection with OURT88/3 isolate providing support for reduced levels of functional CXCL8 in supernatants from Benin 97/1 infected cells.

Although further work is needed to understand the effects of ASFV infection in pigs on host chemokine responses, the changes induced in infected macrophages *in vitro* are likely to be relevant in understanding and predicting responses *in vivo*. It would be of interest to extend the *in vitro* studies to include mixed cultures of macrophages and autologous lymphocytes to investigate to evaluate possible effects of bystander non-infected cell populations. The increased expression of key inflammatory chemokines in macrophages infected with the low virulence isolate OURT88/3 compared to high virulence isolate Benin 97/1 may be important for the activation of a protective immune response in pigs immunised with this isolate.

## Appendix A Supplementary data

Table S1 Summary of primers and reaction conditions used in this study.

Table S2 Functions of chemokines and chemokine receptors changes in mRNA levels detected following ASFV infection.
