# Supplementary material for: Modulation of chemokine and chemokine receptor expression following infection of porcine macrophages with African swine fever virus
Source: Vet Microbiol. 2013 Mar 23;162(2-4):937–43. doi: 10.1016/j.vetmic.2012.11.027 (PMC3605585; doi:10.1016/j.vetmic.2012.11.027)
Supplement: Table S1 — Summary of primers and reaction conditions used in this study. [file mmc1.docx]

Supplementary Table 1 Summary of primers and reaction conditions used in this study.

| Symbol | Accession  Number | Primers | Position | Amplicon  Size (bp) | Primer  Concentration (nM) | Annealing  Temperature (⁰C) | **Primers†** |
| --- | --- | --- | --- | --- | --- | --- | --- |
| CCL2 * | NM_214214  (USDA) | F GCGGCTGATGAGCTACAGAAG  R CCGCGATGGTCTTGAAGATC | 190-210  263-244 | 74bp | 300nM  900nM | 60 | D |
| CCL3L1 | NM_001009579 | F CTTCCTCGCAAATTCGTAGC  R GCATTCAGCTCCAGGTCAG | 127-146  278-260 | 152bp | 100nM  200nM | 60 | D |
| CCL4 | NM_213779 | F GCAAGACCATGAAGCTCTGC  R AAGCTTCCGCACGGTGTATG | 21-40  157-138 | 137bp | 100nM  200nM | 60 | D |
| CCL5 | NM_001129946 | F ATCAGCCTCCCCATATGCCT  R CCGCACCCATTTCTTCTCTG | 123-142  306-287 | 184bp | 100nM  200nM | 58 | D |
| CCR1* | NM_001001621  (USDA) | F AACCCCGTGCCAGAAGGTA  R GCCGATCAGGCCAATGAC | 430-448  520-503 | 91bp | 900nM  900nM | 60 | S |
| CCR5* | NM_001001618  (USDA) | F GCACAGCTCAGCTGGTCAGA  R CGTCGTTTGATAATCCATTTTGC | 17-36  104-82 | 88bp | 900nM  900nM | 60 | D |
| CCR7 | NM_001001532 | F TTGTCATTTTCCAGGTGTGC  R GTACATGACCGGGAGGAACC | 98-117  246-227 | 149bp | 300nM  300nM | 63 | D |
| CXCL2 | NM_001001861 | F CCGTGCAAGGAATTCACCTC  R TGCGGGGTTGAGACAAACTT | 176-195  300-281 | 125bp | 100nM  200nM | 60 | D |
| CXCL8 | NM_213867 | F CAGAACTTCGATGCCAGTGC  R CCTTCTGCACCCACTTTTCC | 172-191  345-326 | 174bp | 100nM  200nM | 56 | D |
| CXCL10 | NM_001008691 | F CCCACATGTTGAGATCATTGC  R CATCCTTATCAGTAGTGCCG | 239-259  406-387 | 168bp | 100nM  200nM | 60 | D |
| CXCR3L | XM_003360358 | F CATGGTCCCTGAGATGAGC  R GCTGAGGCTGAAGTCCTGC | 68-86  212-194 | 145bp | 300nM  300nM | 60 | D |
| CXCR4 | NM_213773 | F GGTTCCGTATATTCACTTCAGA  R ACAGTGGGCAGAAAGATTCG | 8-29  134-115 | 127bp | 300nM  300nM | 63 | D |

Primer references * (USDA) **(Ref)

†Localisation of forward and reverse primers in different exons (D) or the same exon (S). ‡ Single exon gene
