# Supplementary material for: Modulation of chemokine and chemokine receptor expression following infection of porcine macrophages with African swine fever virus
Source: Vet Microbiol. 2013 Mar 23;162(2-4):937–43. doi: 10.1016/j.vetmic.2012.11.027 (PMC3605585; doi:10.1016/j.vetmic.2012.11.027)
Supplement: Table S2 — Functions of chemokines and chemokine receptors changes in mRNA levels detected following ASFV infection. [file mmc2.docx]

Supplementary Table 2 Functions of chemokines and chemokine receptors changes in mRNA levels detected following ASFV infection

| **A Chemokine** | **Change in mRNA levels in ASFV-infected cells** | **Receptor** | **Major target cells showing chemotaxis** |
| --- | --- | --- | --- |
| CCL2 | reduced | CCR2 | monocytes, memory T cells, NK cells, basophils |
| CCL3L1 | reduced | CCR1, CCR3, CCR5 | Monocytes, T cells, NK cells, basophils, eosinophils, dendritic cells, haematopoietic progenitors |
| CCL4 | Increased* | CCR5 | Monocytes, T cells, dendritic cells, NK cells, haematopoietic progenitors |
| CCL5 | unchanged | CCR1, CCR5 | T cells, eosinophils, basophils, NK cells, dendritic cells |
| CXCL2 | reduced | CXCR4, CXCR2 | Neutrophils, endothelial cells |
| CXCL8 | Increase OURT88/3, decrease Benin 97/1* | CXCR1, CXCR2 | Neutrophils, basophils, endothelial cells, T cells |
| CXCL10 | Increase* | CXCR3 | T cells |
| **B Chemokine Receptor** |  | **Chemokine ligand** | **Receptor expressing cells** |
| CCR1 | reduced | CCL3, CCL5, CCL7, CCL8, CCL14, CCL15, CCL23 | Monocytes, macrophages, T cells, NK cells, neutrophils, dendritic cells |
| CCR5 | reduced | CCL3, CCL4, CCL5 | Th1 cells, NK cells, monocytes, immature dendritic cells, thymocytes |
| CCR7 | increased | CCL19, CCL21 | T cells, B cells, dendritic cells, |
| CXCR3L | reduced* | CXCL9, CXCL10, CXCL11 | T cells, B cells, NK cells, monocytes macrophages, mesangial cells, smooth muscle cells, endothelial cells |
| CXCR4 | reduced | CXCL12 | Haematopoietic progenitors, T cells, Immature DCs, monocytes, B cells, PMNs, platelets, astrocytes, endothelial cells/ |
